# The major trimeric antenna complexes serve as a site for qH-energy dissipation in plants

**DOI:** 10.1016/j.jbc.2022.102519

**Published:** 2022-09-22

**Authors:** Pierrick Bru, Collin J. Steen, Soomin Park, Cynthia L. Amstutz, Emily J. Sylak-Glassman, Lam Lam, Agnes Fekete, Martin J. Mueller, Fiamma Longoni, Graham R. Fleming, Krishna K. Niyogi, Alizée Malnoë

**Affiliations:** 1 Department of Plant Physiology, Umeå Plant Science Centre (UPSC), Umeå University, Umeå, Sweden; 2 Department of Chemistry, University of California, Berkeley, California, USA; 3 Molecular Biophysics and Integrated Bioimaging Division (Formerly Physical Biosciences Division), Lawrence Berkeley National Laboratory, Berkeley, California, USA; 4 Kavli Energy Nanoscience Institute, Berkeley, California, USA; 5 School of Energy, Materials and Chemical Engineering, Korea University of Technology and Education, Cheonan, Chungnam, Republic of Korea; 6 Department of Plant and Microbial Biology, Howard Hughes Medical Institute, University of California, Berkeley, California, USA; 7 Graduate Group in Biophysics, University of California, Berkeley, California, USA; 8 Julius-von-Sachs-Institute, Biocenter, Pharmaceutical Biology, University of Wuerzburg, Wuerzburg, Germany; 9 Institute of Biology, University of Neuchâtel, Neuchâtel, Switzerland

**Keywords:** photosynthesis, energy dissipation, nonphotochemical quenching qH, *Arabidopsis thaliana*, light-harvesting complexes, time-resolved fluorescence, CRISPR–Cas9, abiotic stress, Cas9, CRISPR-associated nuclease 9, Chl, chlorophyll, α-DM, α-dodecyl maltoside, *F*_m_, maximum fluorescence, HHMI, Howard Hughes Medical Institute, HL, high light, LCNP, lipocalin in the plastid, Lhcb, light-harvesting complex, NPQ, nonphotochemical quenching, OE, overexpressor, PSII, photosystem II, PsbS, photosystem II subunit S, ROQH1, relaxation of qH 1, SOQ1, suppressor of quenching 1, TBST, Tris-buffered saline with Tween-20

## Abstract

Plants and algae are faced with a conundrum: harvesting sufficient light to drive their metabolic needs while dissipating light in excess to prevent photodamage, a process known as nonphotochemical quenching. A slowly relaxing form of energy dissipation, termed qH, is critical for plants’ survival under abiotic stress; however, qH location in the photosynthetic membrane is unresolved. Here, we tested whether we could isolate subcomplexes from plants in which qH was induced that would remain in an energy-dissipative state. Interestingly, we found that chlorophyll (Chl) fluorescence lifetimes were decreased by qH in isolated major trimeric antenna complexes, indicating that they serve as a site for qH-energy dissipation and providing a natively quenched complex with physiological relevance to natural conditions. Next, we monitored the changes in thylakoid pigment, protein, and lipid contents of antenna with active or inactive qH but did not detect any evident differences. Finally, we investigated whether specific subunits of the major antenna complexes were required for qH but found that qH was insensitive to trimer composition. Because we previously observed that qH can occur in the absence of specific xanthophylls, and no evident changes in pigments, proteins, or lipids were detected, we tentatively propose that the energy-dissipative state reported here may stem from Chl–Chl excitonic interaction.

Photosynthetic organisms possess pigment–protein antenna complexes, which can switch from a light-harvesting state to an energy-dissipating state (1, 2). This switching capability regulates how much light is directed toward photochemistry and ultimately how much carbon dioxide is fixed by photosynthesis (3). The fine-tuning of light energy usage is achieved at the molecular level by proteins that act at or around these pigment–protein complexes (4). Understanding the regulatory mechanisms involved in the protection against excess light, or photoprotection, has important implications for engineering optimized light-use efficiency in plants (5) and thereby increasing crop productivity when light reactions are limiting such as upon transition from sun to shade (6, 7, 8) and/or tolerance to photo-oxidative stress in suboptimal environments (9).

Nonphotochemical quenching (NPQ) processes protect photosynthetic organisms by safely dissipating excess absorbed light energy as heat and is assessed as a decrease of chlorophyll (Chl) fluorescence (10). Indeed absorbed light energy by Chl can fuel photosynthetic reaction (photochemistry), be re-emitted as heat or as fluorescence (11). Upon blocking photochemistry using a saturating pulse of light, maximum fluorescence (*F*_m_) is measured and inversely correlated with the amount of energy dissipated by NPQ (12, 13). Several NPQ mechanisms have been described and classified based on their recovery kinetics and/or molecular players involved (14, 15, 16): qE, qZ, qH, qI, qT with the letter “q” referring to quenching, that is, decrease of fluorescence, followed by a letter specifying the mechanism. In plants, the rapidly reversible NPQ (relaxes within minutes), or flexible energy dissipation mode, qE, relies on ΔpH, the protein PsbS, and the xanthophyll pigment zeaxanthin (17). The slowly reversible NPQ (relaxes within hours to days), or sustained energy dissipation mode, includes several mechanisms such as qZ (zeaxanthin dependent, ΔpH independent (18)), qH (see later and Ref. (15) for a review), and qI (due to photosystem II [PSII] reaction center subunit D1 photoinactivation (19), which can be reversed by D1 repair (20)). Energy redistribution through qT is due to state transition, the movement of antenna phosphorylated by the kinase STN7 (21).

We have recently uncovered, using chemical mutagenesis and genetic screens in *Arabidopsis thaliana*, several molecular players regulating a slowly reversible NPQ mechanism, which we named qH (22, 23, 24, 25). qH requires the plastid lipocalin (LCNP) (25) for its induction or for its activation, is negatively regulated by suppressor of quenching 1 (SOQ1) (23, 26), and is inactivated by relaxation of qH 1 (ROQH1) (22). Importantly, qH is independent of PsbS, ΔpH, xanthophyll pigments, and phosphorylation by STN7 (23, 25). Strikingly, when qH is constitutively active in a *soq1 roqh1* mutant, plants are severely light limited and display a stunted phenotype (22). If qH cannot occur (as in an *lcnp* mutant), a higher amount of lipid peroxidation is observed, and plants are severely light damaged under stress conditions such as cold temperature and high light (HL) (25, 27). Our present working hypothesis is that, under stress conditions, LCNP binds or modifies a molecule in the vicinity of or within the antenna proteins, thereby triggering a conformational change that converts antenna proteins from a light-harvesting to a dissipative state.

In WT *Arabidopsis* plants, qH occurs in response to cold and HL (25), whereas the *soq1* mutant can display qH under nonstress conditions upon a 10 min HL treatment (23). In plants, the peripheral antenna of PSII is composed of pigment-binding, light-harvesting complex (Lhcb) proteins, which are divided into minor subunits (Lhcb4, Lhcb5, Lhcb6, or CP29, CP26, CP24, respectively) present as monomers and major subunits (Lhcb1, Lhcb2, and Lhcb3) also referred to as LHCII, forming heterotrimeric and homotrimeric complexes associated to PSII in a strongly, moderately, or loosely bound manner (28, 29). The pigments associated with the major and minor antenna complexes include Chls *a* and *b* and xanthophylls, such as lutein, violaxanthin, zeaxanthin, and neoxanthin (30). The mutant *chlorina1* does not accumulate trimeric Lhcbs because it lacks Chl *b*, but it does accumulate some monomeric Lhcbs with Chl *a* only (31). qH is no longer observed in the double mutant *soq1 chlorina 1* (25), indicating that qH may require the trimeric antenna and/or Chl *b*. Here, we investigated whether qH remained active upon isolation of thylakoids or photosynthetic subcomplexes with aim to narrow down the location of the qH quenching site and characterize its properties. We measured the Chl fluorescence lifetimes of intact leaves, isolated thylakoids, and isolated complexes from plants (WT and several mutants relating to qH) exposed to nonstress or stress conditions with active or inactive qH. Isolation of partly quenched LHCII directly from thylakoid membranes with active qH showed that qH can occur in the major trimeric LHCII complexes. Through genome editing and genetic crosses, we further demonstrated that qH does not rely on a specific major Lhcb subunit, suggesting that qH is not because of specific amino acid variation among Lhcb1, Lhcb2, and Lhcb3 (such as phosphorylation in Lhcb1 and Lhcb2 or the presence of cysteine in Lhcb2.3 or aromatic residues in Lhcb3) and/or that compensation from other major Lhcb proteins may occur. Prior to this work, only a few studies had reported a quenched conformation of isolated LHCII trimers, and in contrast to the native isolation reported here, quenching was achieved *in vitro*, after full solubilization of LHCII (32, 33). Successful isolation of natively quenched LHCII paves the way for revealing its molecular origin.

## Results

### Chl fluorescence lifetimes are decreased by qH in leaves and thylakoids

**Figure 1:**
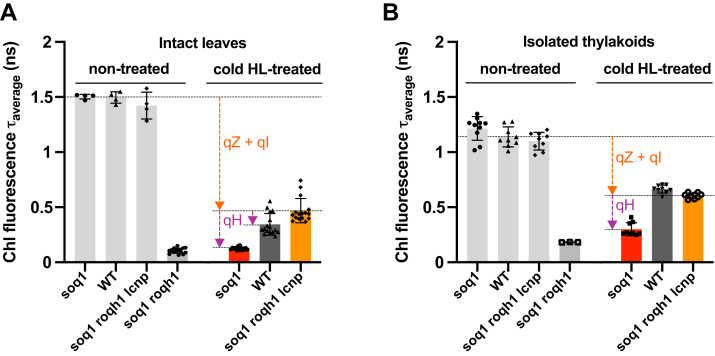
**qH decreases chlorophyll fluorescence lifetimes of leaves and thylakoids.** Average fluorescence lifetime (τ_average_) of intact leaves (*A*) or crude thylakoid membrane isolated (*B*) from nontreated plants *soq1*, WT, *soq1 roqh1 lcnp*, and *soq1 roqh1* or cold and high light (cold HL)–treated plants *soq1*, WT, and *soq1 roqh1 lcnp* for 6 h at 6 °C and 1500 μmol photons m^−2^ s^−1^. qE is relaxed by dark acclimating for 5 min before each measurement (for nontreated isolated thylakoids, dark acclimation of detached leaves overnight prior to thylakoid extraction). Excitation at 420 nm and emission at 680 nm. Data represent means ± SD (intact leaves, nontreated, n = 4 plant individuals and n = 17 for *soq1 roqh1*, cold HL-treated, n = 14–16; isolated thylakoids, n = 3–6 technical replicates from two independent biological experiments each with n ≥ 5 plants; one biological experiment for *soq1 roqh1*). NPQτ values are determined based on $NPQ\tau =\frac{\tau_{\mathrm{avg},\mathrm{nontreated}}-\tau_{\mathrm{avg},\mathrm{cold}\;\mathrm{HL}-\mathrm{treated}}}{\tau_{\mathrm{avg},\mathrm{cold}\;\mathrm{HL}-\mathrm{treated}}}$. NPQτ of leaves from cold HL-treated *soq1*, WT, and *soq1 roqh1 lcnp* are 11, 3.3, and 2, respectively, and of isolated thylakoids 3.1, 0.7, and 0.8. NPQ, nonphotochemical quenching.

Previously, we demonstrated that qH is induced by a cold and HL treatment on whole plants of *Arabidopsis* in mutants and importantly also in WT. We found that the amount of NPQ measured by Chl fluorescence imaging can reach a high level, approximately 12 in the *soq1* mutant, and this induction of NPQ is LCNP dependent as it does not occur in the *soq1 lcnp* double mutant (25). We also observed constitutive qH from nontreated plants in the *soq1 roqh1* double mutant, which displayed *F*_m_ values ∼85% lower than WT or *soq1 roqh1 lcnp*, indicating a high NPQ yield (22). To ascertain that qH under stress condition such as cold and HL, or in the double mutant *soq1 roqh1*, was due to a decrease in Chl excited-state lifetime, we measured fluorescence lifetime *via* time-correlated single photon counting on both intact leaves and thylakoids isolated from plants cold and HL treated or nontreated. Here, we used the laser at a saturating light intensity to close PSII reaction centers so that differences in lifetime can be attributed to NPQ (34). Strikingly, nontreated *soq1 roqh1* indeed displayed a decreased amplitude-weighted average fluorescence lifetimes (τ_avg_) compared with controls (Fig. 1, *light gray bars*), with a much shorter value in both leaves (∼0.1 ns *versus* ∼1.5 ns) and thylakoids (∼0.2 ns *versus* ∼1.1 ns). These data unequivocally show that LCNP-dependent NPQ, qH, promotes a Chl de-excitation pathway, which remains active upon isolation of thylakoid membranes.

Next, we exposed plants to a 6 h cold and HL treatment (6 °C and 1500 μmol photons m^−2^ s^−1^) followed by dark acclimation for 5 min to relax qE. During this treatment, qH is induced and so is qZ as zeaxanthin accumulates (de-epoxidation state value of approximately 0.7 [stress] *versus* 0.05 [nonstress] in all lines (22, 25)); the remaining slowly relaxing quenching processes are grouped under the term qI and are in part due to photoinactivation of PSII. In Figure 1, colored bars show that the cold and HL treatment on plants results in a decreased Chl excited-state lifetime in both leaves and isolated thylakoids. Interestingly, cold and HL-treated *soq1* leaves displayed τ_avg_ values similar to nontreated *soq1 roqh1* indicating that this stress treatment triggers a fully active qH state in *soq1*. WT leaves displayed an intermediate τ_avg_ value between active qH (*soq1*) and inactive qH (*soq1 roqh1 lcnp*), further establishing the occurrence, and physiological relevance, of qH in promoting energy dissipation under abiotic stress (Fig. 1*A*). The calculated NPQτ derived from the τ_avg_ values were all in agreement with the NPQ values measured by pulse amplitude–modulated Chl fluorescence (22, 25) with 11, 3.3, and 2 for *soq1*, WT, and *soq1 roqh1 lcnp*, respectively; *soq1 lcnp* was not measured here as its phenotype is similar to *soq1 roqh1 lcnp*. We also measured the fluorescence lifetime from leaves of *soq1 npq4 roqh1* (lacks SOQ1, PsbS, and ROQH1), *soq1 roqh1* ROQH1 overexpressor (OE), and several mutant alleles of *roqh1* and *soq1 roqh1* (Fig. S1 and Table S1). The τ_avg_ values, and calculated NPQτ, further highlighted that qH is independent of PsbS (*soq1 npq4 roqh1* has a low τ_avg_ in a similar range to *soq1 roqh1*, ∼60 ps *versus* 130 ps) and that ROQH1 is required for relaxation of qH (NPQτ of *roqh1* mutant and *soq1 roqh1* ROQH1 OE, respectively, higher and lower than WT).

Of note, in thylakoids isolated from cold and HL-treated plants, the τ_avg_ values were overall higher than observed in intact leaves, and the difference in τ_avg_ between WT and *soq1 roqh1 lcnp* was no longer apparent (Fig. 1*B*), whereas the τ_avg_ values of isolated thylakoids from nontreated plants without qH (*soq1*, WT, and *soq1 roqh1 lcnp*) were all lower than observed in intact leaves. Therefore, possible changes in, for example, membrane macro-organization, protein content, or complexes occurring during thylakoid isolation have an opposite effect on fluorescence lifetime depending on the starting state (active or inactive NPQ) for reasons we cannot explain. We probed the release of Chl fluorescence by step solubilization of thylakoid membrane preparation (Fig. S2), and it showed that qH is partly due to protein–protein and lipid–protein interactions in the membrane (cold and HL, Q_m_ *soq1* higher than *soq1 roqh1 lcnp*) and due to pigment–protein interactions (Q_pi_ also higher), which may explain the lower NPQτ of *soq1* thylakoids compared with leaves (and longer τ_avg_ of *soq1 roqh1* thylakoids compared with leaves) as some of these interactions may have been lost during thylakoid preparation. Yet, although smaller, the retention of active qH in *soq1* thylakoids offered a unique opportunity to explore whether quenched photosynthetic subcomplexes could be isolated.

### Isolated LHCII trimers from plants with active qH are quenched

**Figure 2:**
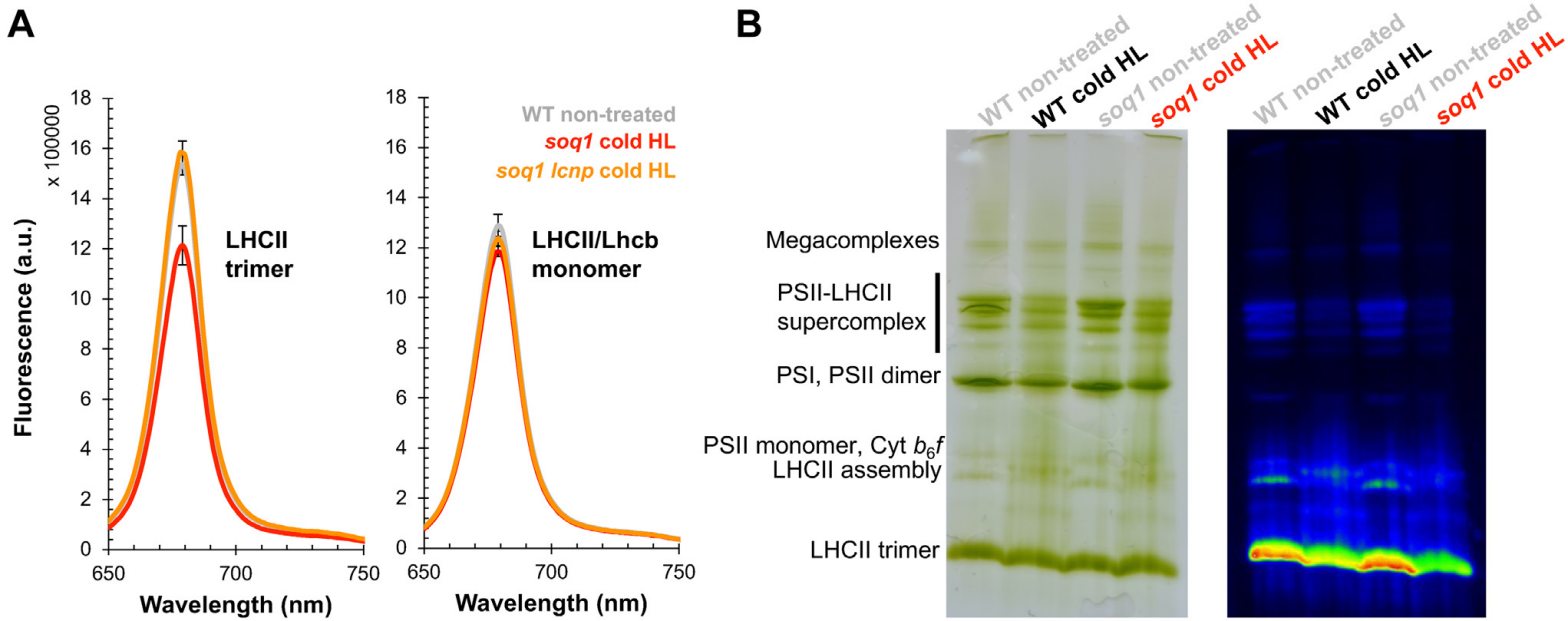
**Isolated LHCII trimers from plants with active qH are quenched.***A*, room temperature fluorescence spectra of isolated LHCII trimer (*left*) and LHCII/Lhcb monomer (*right*) pooled fractions from nontreated WT (*gray*) and cold and high light (HL) (cold HL)-treated *soq1* (*red*) and *soq1 lcnp* (*orange*) (see Fig. S3 for gel filtration experiments and peak annotation from which fractions were pooled). Fluorescence emission from 650 nm to 750 nm from samples diluted at same chlorophyll concentration (0.1 μg ml^−1^) with excitation at 625 nm, with maxima at 679 nm for all samples. Data represent means ± SD (n = 3 technical replicates from biological replicate 3 with n = 8 plants). Representative from three independent biological experiments is shown (see Fig. S4, *A* and *B* for biological replicates 1 and 2). *B*, thylakoids were extracted from WT and *soq1* plants (n = 3 individuals of each) grown under standard conditions (*gray*) or cold HL-treated for 10 h (*black* and *red*) with NPQ values of respectively 3 ± 1 and 11 ± 1, solubilized in 1% α-DM, and separated by clear native PAGE on a 3 to 12% gel. 10 μg Chl were loaded per lane. Gel image (*left*) and chlorophyll fluorescence image (*right*). The composition of the major bands is indicated based on the study of Ref. (68). Representative from two independent biological experiments (with n ≥ 3 plants) is shown (see Fig. S4*C* for LHCII trimer pigment–protein band and chlorophyll fluorescence quantification).α-DM, α-dodecyl maltoside; Lhcb, light-harvesting complex; NPQ, nonphotochemical quenching.

Next, we tested whether we could observe qH in a specific isolated pigment–protein complex. The lines *soq1* (active qH) and *soq1 lcnp* (inactive qH) were chosen for this purpose (*soq1 roqh1* and *soq1 roqh1 lcnp* could have been used, but *soq1 roqh1* plants are much smaller because of light limitation by constitutive active qH). Plants were treated with cold and HL for 6 h at 6 °C and 1500 μmol photons m^−2^ s^−1^ followed by dark acclimation for 5 min to relax qE. Thylakoids were isolated, solubilized, and fractionated by gel filtration to separate complexes based on their size. The separation profiles of photosynthetic complexes were similar for *soq1* and *soq1 lcnp* (Fig. S3*A*). Fractions corresponding to PSII–LHCII megacomplexes, supercomplexes, PSI–LHCI supercomplexes, PSII core dimer, LHCII trimer, and LHCII/Lhcb monomer (containing both major and minor antenna, see later) as well as smaller fractions (peaks 7 and 8) were collected, and their relative fluorescence yield was measured by video imaging (Fig. S3*B*). The LHCII trimer fraction clearly displayed a lower relative fluorescence yield with active qH. Room-temperature fluorescence spectra were measured at the same low Chl concentration (0.1 μg ml^−1^) to prevent reabsorption and with excitation at 625 nm (isosbestic point) to excite both Chls *a* and *b* equally; the Chl *a*/*b* ratio is similar between the compared samples so absorption at 625 nm should be equal. Complexes from nontreated WT were isolated for reference; material came from plants grown under standard light conditions. The LHCII trimer fraction displayed a relative fluorescence yield at 680 nm that was on average 24% ± 8% lower with active qH compared with inactive qH and WT reference, whereas the LHCII/Lhcb monomer fraction displayed no significant differences among samples (Figs. 2*A* and S4, *A* and *B*). A complementary approach separating pigment–protein complexes following solubilization by clear native-PAGE further evidenced that qH is active in isolated LHCII trimers (Figs. 2*B* and S4*C*). These results suggest that qH occurs at least partly in the LHCII trimer and remains active even after isolation of the solubilized protein complex.

**Figure 3:**
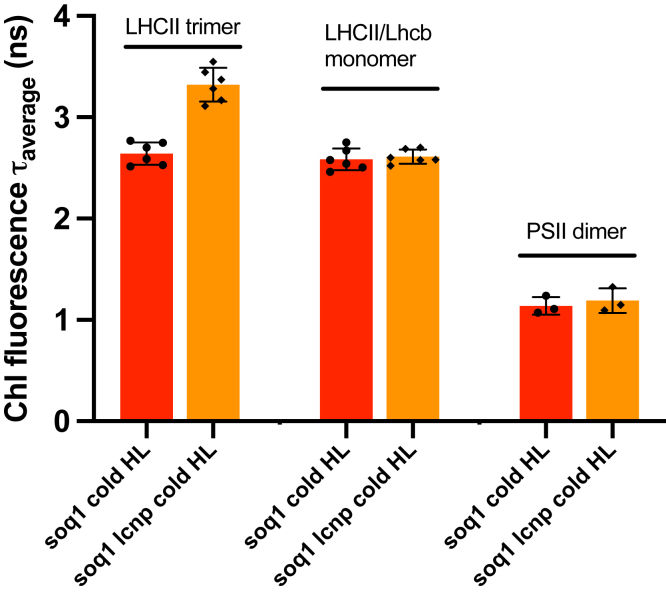
**qH decreases chlorophyll fluorescence lifetimes of isolated LHCII trimers.** Average fluorescence lifetime (τ_avg_) of LHCII trimer, LHCII/Lhcb monomer, and PSII dimer isolated from cold HL-treated *soq1* (*red*) and *soq1 lcnp* (*orange*) plants. Data represent means ± SD (n = 3 technical replicates from two independent biological experiments each with n = 8 plants). HL, high light; Lhcb, light-harvesting complex; PSII, photosystem II.

We measured the Chl fluorescence lifetimes of LHCII trimer, LHCII/Lhcb monomer, and PSII dimer fractions. We observed in the active qH LHCII trimer fraction a ∼20% shorter τ_avg_ compared with that of inactive qH (∼2.6 ns for *soq1 versus* ∼3.3 ns for *soq1 lcnp*) in agreement with the ∼20% decrease in relative fluorescence yield (Fig. 3); for reference, nontreated WT LHCII τ_avg_ is ∼3.1 ns (Table S2). No differences in fluorescence lifetimes caused by qH were detected in either the LHCII/Lhcb monomer or PSII dimer fractions. These results unambiguously demonstrate that qH promotes a Chl de-excitation pathway in the trimeric antenna and is distinct from qI. In our previous work, the question persisted whether qH was antenna dependent as we had not shown direct evidence of quenching in the antenna.

### No evident changes in pigment, lipid, and protein content of quenched LHCII

We examined the pigment, lipid, and protein content by HPLC, LC–MS, and SDS-PAGE, respectively, to investigate which molecular changes may be at the origin of the qH-energy dissipative state in the trimeric antenna. There were no apparent differences in pigment composition (Fig. S5*A*) or abundance (Fig. S5*B*) in LHCII trimers from active or inactive qH. Composition of the main chloroplastic lipids in LHCII trimer, LHCII/Lhcb monomer, or thylakoid extracts indicated no significant differences (Figs. S6 and S7); the distribution of thylakoid lipids is in line with the literature (35). Of note, the 6 h cold and HL treatment did not alter the lipid profile significantly. The protein content was also similar in LHCII trimer from active or inactive qH (with an equivalent low amount of minor monomeric Lhcb4), and there were no visible additional protein bands or size shifts (Fig. S8). Investigation of possible post-translational modifications of amino acid residues by protein mass spectrometry will be the subject of future work (preliminary exploration did not show evident changes). We observed LHCII subunits in the monomer fractions (probed with anti-Lhcb2), hence the “LHCII/Lhcb” name, with a higher content in the cold and HL-treated samples compared with nontreated WT; this could be due to monomerization of trimers during the cold and HL treatment.

### qH does not rely on a specific major LHCII subunit

**Figure 4:**
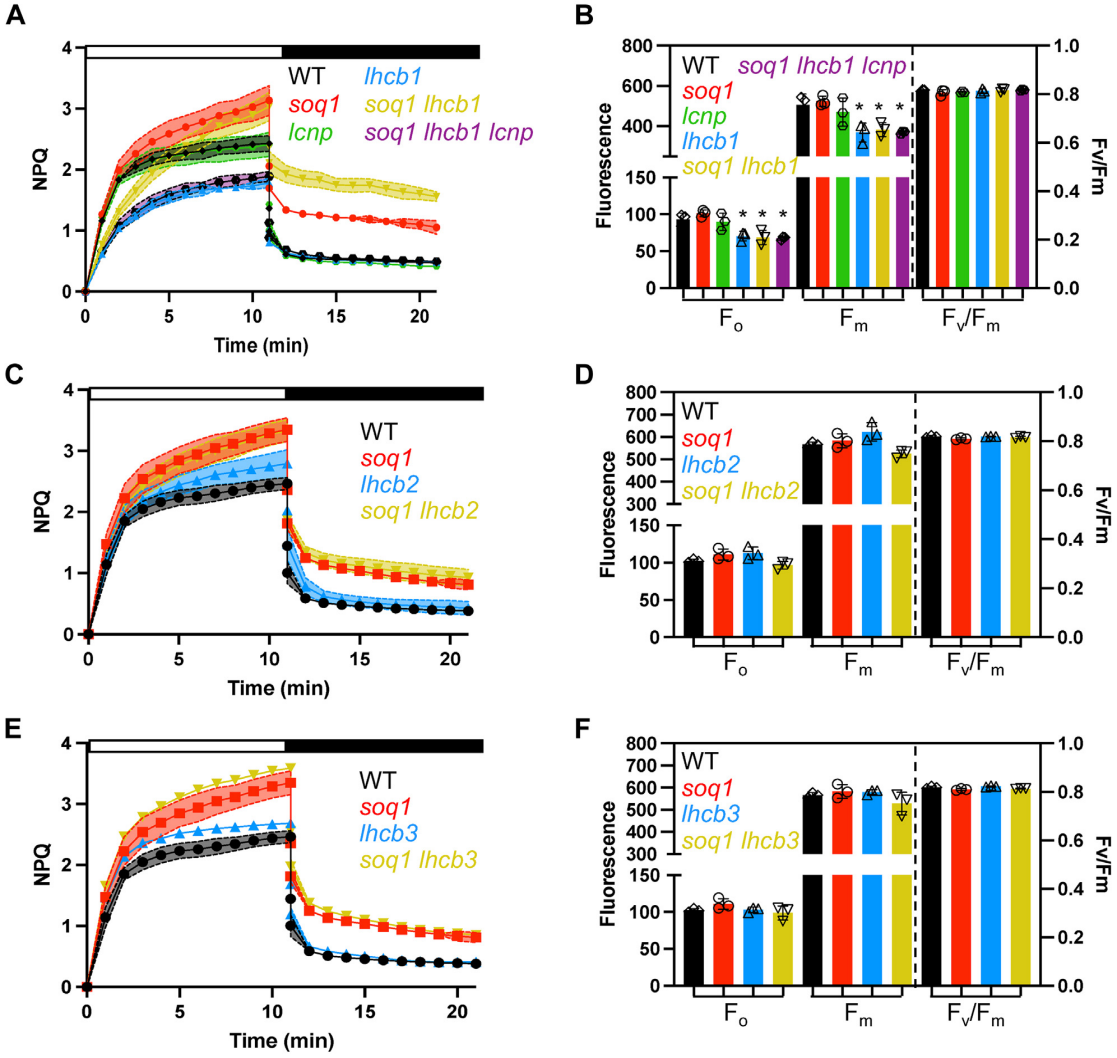
**qH does not rely on a specific major Lhcb.***A*, *C*, and *E*, NPQ kinetics of WT, *soq1*, *lcnp*, *lhcb1*, *soq1 lhcb1*, *soq1 lhcb1 lcnp*, *lhcb2*, *soq1 lhcb2*, *lhcb3*, and *soq1 lhcb3* 4-week-old plants grown at 120 μmol photons m^−2^ s^−1^ dark acclimated for 20 min. Induction of NPQ at 1200 μmol photons m^−2^ s^−1^ (*white bar*) and relaxation in the dark (*black bar*). *B*, *D*, and *F*, photosynthetic parameters *F*_o_, *F*_m,_ and *F*_v_/*F*_m_ of the same plants. Tukey's multiple comparisons test shows that *lhcb1*, *soq1 lhcb1*, and *soq1 lhcb1 lcnp* are statistically different from WT for *F*_o_ (*p* = 0.0359, *p* = 0.0222, and *p* = 0.0171, respectively) and *F*_m_ (*p* = 0.0245, *p* = 0.0482, and *p* = 0.0257, respectively). Small significant difference in *F*_m_ with *p* = 0.0111 for *lhcb2* and *soq1 lhcb2* was not observed in two other biological experiments. Data represent means ± SD (*n* = 3 plant individuals).

Having gained the knowledge that qH partly occurs in the LHCII trimer, the next question was whether a specific LHCII subunit would be required, and this may provide a hint to the molecular origin of qH. We used genetic crosses together with genome editing to combine the *soq1* mutation with mutations in *LHCII* genes. The *soq1* mutant was crossed to *lhcb1* or *lhcb2* mutant lines generated by CRISPR–Cas9–mediated genome editing or to the transfer DNA insertional mutant *lhcb3*. The dissection of a putative specific LHCII quenching site is no small feat as there are five *LHCB1* genes (*LHCB1.1*, *1.2*, and *1.3* are neighboring genes, so are *LHCB1.4* and *1.5*), three *LHCB2* genes (*LHCB2.1* and *2.2* are neighboring genes, and *LHCB2.3*), and one *LHCB3* gene. Three “loci” are therefore segregating upon generating the sextuple *soq1 lhcb1* or the quadruple *soq1 lhcb2* mutants. We genotyped the lines by PCR and confirmed lack of specific LHCII isoforms by immunoblot analysis (Fig. S9*A*). In all three mutant combinations, *soq1 lhcb1*, *soq1 lhcb2*, or *soq1 lhcb3*, additional quenching compared with the respective *lhcb* mutant controls was observed (Fig. 4, *A*, *C* and *E*), which suggests that qH does not require a specific LHCII isoform; of note, NPQ can be compared between *lhcb* and *soq1 lhcb* mutants as they possess similar *F*_m_ values (Fig. 4, *B*, *D* and *F*). In the case of *soq1 lhcb1*, only few trimers should be remaining (36, 37), but the NPQ difference between *lhcb1* and *soq1 lhcb1* is higher than between WT and *soq1*. We therefore generated the *soq1 lhcb1 lcnp* to ensure that all additional quenching in *soq1 lhcb1* is qH (*i.e.*, LCNP-dependent). The NPQ kinetics of *soq1 lhcb1 lcnp* and *lhcb1* were similar, which confirms that this additional quenching is qH and is enhanced when Lhcb1 is lacking (Figs. 4*A* and S9*B*).

## Discussion

Here, we have characterized the Chl fluorescence lifetimes of leaves, thylakoids, and isolated photosynthetic subcomplexes directly from *Arabidopsis* plants with active or inactive qH. We demonstrated that qH promotes a Chl de-excitation pathway, which remains active upon isolation of thylakoid membranes (Fig. 1) and isolation of LHCII trimers (Figs. 2 and 3) (see also summary in Table S2), but the effect is much smaller in isolated trimers than in leaves or thylakoids likely because of a large proportion of unquenched LHCII that dominates the ensemble.

The lifetime of nontreated *soq1 roqh1* leaves with constitutive activation of qH is among the shortest ever observed with only ∼0.1 ns (for comparison, a qE-induced state has a lifetime of ∼0.5 ns (38) or the astaxanthin-synthesizing tobacco ∼0.6 ns (39)) and confirms the stunted growth of *soq1 roqh1* (22) as being due to impaired light-harvesting function. Cold and HL-induced qH active plants display a short average fluorescence lifetime in leaves, similar to *soq1 roqh1* (∼0.1 ns), and the lifetime increases in thylakoids (∼0.4 ns) and is higher in isolated LHCII trimers (∼2.6 ns). Whereas intact leaves and thylakoids of treated *soq1* with active qH showed a large amplitude of a rapidly decaying fluorescence component (A1 > 50%) and a small amplitude of a long-lived component (A3 < 10%), this trend was much less apparent at the level of isolated LHCII but yet remained true relative to *soq1 lcnp* (Table S3). Therefore, the long average fluorescence lifetime of LHCII isolated from treated *soq1* (∼2.6 ns) is likely because of a decreased amplitude of rapid components (A1 ∼ 18%) and increased amplitude of slow components (A3 ∼ 73%) in the LHCII fraction relative to thylakoids. These results indicate that qH may relax during isolation of thylakoids or photosynthetic complexes (the latter takes about 8 h from leaf material collection to fluorescence lifetime measurements) and that the trimer fraction is a heterogenous population of quenched and unquenched LHCII. Furthermore, fluorescence lifetimes of pigment–protein complexes largely depend on their local environment, for example, detergent or proteoliposome (40, 41, 42), and comparison of LHCII in detergent micelles *versus* membrane nanodiscs shows that quenching is attenuated by detergent (43). Another possible explanation for the differences in fluorescence lifetimes among sample types is that a preserved membrane macro-organization is required for a full qH response; indeed LH1 and LH2 antenna rings in purple bacteria display a 50% shorter lifetime *in vivo* compared with *in vitro* (44), and similarly, quenching in LHCII is dependent on its membrane environment (45, 46, 47, 48). There could also be a mixed population of trimers with active/inactive qH in an intact leaf, and this would become more evident once isolated (assuming connectivity between trimers is required for full quenching); the resulting average lifetime would thus be an average of an ensemble of LHCII trimers with varying degrees of qH. For qE, it has been modeled that 12% of sites with active NPQ are sufficient to explain WT levels of NPQ (49), and it is feasible that a similar situation could underlie qH. In addition, other quenching sites beyond the LHCII trimers for qH may exist.

Nevertheless, the successful isolation of natively quenched LHCII by qH paves the way for revealing its molecular origin. We have not observed any significant changes in pigment, lipid, or protein content of LHCII trimers with active qH (Figs. S5–S8), and we do not have any evidence for pigment photobleaching and/or oxidized Chl photoproducts, which are accompanied with fluorescence quenching in photodamaged isolated LHCII (50). Also qH is photoprotective as it decreases lipid peroxidation and bleaching of leaves under stress (25), so it is unlikely that the quenched LHCII isolated here is due to more photobleaching. Because previous genetic dissection of qH requirement for xanthophyll pigments found that violaxanthin, zeaxanthin, or lutein is dispensable (23, 25), we tentatively propose that qH may stem from a Chl–Chl excitonic interaction state. Small changes in the conformation of the trimer modifying the protein environment of Chls or their orientation and/or distance with each other could enable qH, and the work reported here will enable to identify this fine-tuning. Such changes of the conformational space of proteins or carotenoids have recently been studied for qE experimentally or through molecular dynamics simulations (51, 52) and highlighted that several conformers would underlie light-harvesting and energy-dissipation states providing a more complex picture than previously thought for NPQ regulation. It could also be that qH is due to altered Chl–amino acid or Chl–hydrophobic molecule interaction; for a recent review of Chl quenching mechanisms, see Ref. (53).

Decreased relative fluorescence yield and lifetime of isolated LHCII trimer (and not of isolated monomers) from plants with active qH indicates that qH likely occurs in the trimeric major antenna and not in the minor antenna (Figs. 2 and 3). This interpretation of the results is assuming a similar relaxation rate between the different subcomplexes during isolation. Next, we will investigate the involvement of the minor antenna in regulating qH. The fractionation method used here results in a pool of LHCII trimers comprising the three types of trimers (strongly, moderately, or loosely bound), and whether qH preferentially occurs in a specific type of trimer remains to be explored. Through genetic crosses, we found that qH does not require a specific LHCII subunit (Fig. 4). LHCII trimers are composed of Lhcb1 (70% of the total LHCII proteins), Lhcb2 (20%), and Lhcb3 (10%), which form homotrimers of Lhcb1, of Lhcb2, or heterotrimers of Lhcb1, Lhcb2, and/or Lhcb3 (54). The degree of conservation between these subunits is high with an amino acid identity of 82% between Lhcb1 and Lhcb2, 78% between Lhcb1 and Lhcb3, and 79% between Lhcb2 and Lhcb3 (55). When a specific LHCII subunit is missing, some compensation by other subunits can occur: in the *lhcb1* CRISPR–Cas9 line, Lhcb2 accumulation is increased (37) but possibly insufficiently to fully explain the high NPQ of *soq1 lhcb1* (Fig. 4*A*). The enhanced qH in *soq1 lhcb1* could be explained by a different organization of photosynthetic complexes that would promote qH formation and/or slow down its relaxation, either in remaining LHCII trimers or elsewhere in the membrane. In the *amiLhcb2* or in *lhcb3* lines, trimers are abundant with an increased accumulation of Lhcb3 (36) or Lhcb1 and Lhcb2 (56), respectively. Therefore, the similar NPQ kinetics between *soq1* and *soq1 lhcb2*, or *soq1 lhcb3*, and the enhanced qH in *soq1 lhcb1* (Fig. 4) indicate that qH does not rely on a specific subunit of the LHCII trimer.

To conclude, we isolated and characterized an energy-dissipative state of the major antenna complex directly from plants with active qH, with physiological relevance to natural conditions. Future work will focus on identifying differences in LHCII trimers that are associated with active qH and elucidation of the photophysical mechanism(s) of qH.

## Experimental procedures

### Plant material and growth conditions

WT *A. thaliana* and derived mutants studied here are of Col-0 ecotype. Mutants from these respective studies were used (only the *soq1-1* and *lcnp-1* alleles were used except for the *soq1 lhcb1 lcnp* line in which *lcnp* mutation was obtained through genome editing): *soq1* (23), *soq1 lcnp* (25), *roqh1-1*, *roqh1-2*, *roqh1-3*, *soq1 roqh1-1*, *roqh1-2*, *roqh1-3*, *soq1 roqh1 ROQH1 OE*, *soq1 roqh1 lcnp*, *soq1 npq4 roqh1* (22), and *lhcb3* (SALK_036200C) (57). For clarity, we refer to the *lhcb1* quintuple mutant affected in all five *LHCB1* genes as “*lhcb1*” (CRISPR–Cas9 edits for *lhcb1.1* [nucleotide (nt) insertion 575_576insA], *lhcb1.2* [nt deletion 575del], *lhcb1.3* [nt insertion 419_420insT], *lhcb1.4* [nt insertion 416_417insT], and *lhcb1.5* [large deletion 413_581del]) and to the *lhcb2* triple mutant affected in all three *LHCB2* genes as “*lhcb2*” (*lhcb2.1* [SALK_005774C], CRISPR–Cas9 edits for *lhcb2.2* [nt insertion 10insA], *lhcb2.3* [nt insertion 93insA]). Mutants *soq1 lhcb1*, *soq1 lhcb2*, *soq1 lhcb3*, and *soq1 lhcb1 lcnp* were generated in this study. Plants were grown on soil (Sunshine Mix 4/LA4 potting mix; Sun Gro Horticulture Distribution [Berkeley], 1:3 mixture of Agra-vermiculite “yrkeskvalité K-JORD” provided by RHP and Hasselfors garden, respectively) under a 10/14 h light/dark photoperiod at 120 μmol photons m^−2^ s^−1^ at 21 °C, referred to as standard conditions (Berkeley) or 8/16 h at 150 μmol photons m^−2^ s^−1^ at 22 °C/18 °C for 5 to 6 weeks, or seeds were surface sterilized using 70% ethanol and sown on agar plates (0.5× Murashige and Skoog Basal Salt Mixture, Duchefa Biochemie, with pH adjusted to 5.7 with KOH) placed for 1 day in the dark at 4 °C, grown for 3 weeks with 12/12 h at 150 μmol photons m^−2^ s^−1^ at 22 °C, and then transferred to soil. For the cold and HL treatment, plants or detached leaves were placed for 6 h at 6 °C and at 1500 μmol photons m^−2^ s^−1^ using a custom-designed LED panel built by JBeamBio with cool white LEDs BXRA-56C1100-B-00 (Farnell). Light bulbs used in growth chambers are cool white (4100K) from Philips (F25T8/Tl841 25W) for plants grown on soil and from General Electric (F17T8/SP41 17W) for seedlings grown on agar plates. A biological replicate, also referred to as biological experiment, represents a separate batch of several plant individuals grown at independent times. A technical replicate is an independent measurement performed on different aliquots from the same sample.

### Genetic crosses, genome editing, and genotyping primers

Genetic crosses were done using standard techniques (58). Genome editing assisted by CRISPR–Cas9 was used to generate *lhcb1* and *lhcb2* and following the procedure described (59, 60) for the *soq1 lhcb1 lcnp* line. The mutant background *soq1 lhcb1* was used to generate the *soq1 lhcb1 lcnp* line using four single guide (sgRNA) targeting *AtLCNP* exon 1 (CTTGTTGAAGTGGCAGCAGG), exon 3 (CTCACGTTACTGTCAGAAGA), exon 4 (TGACATCATAAGGCAACTTG), and exon 5 (TCAGTCACTTCACAGTCCTG) designed using the online tool CHOPCHOP (61) and further ranked for efficiency score with E-CRISP (62) for the two sgRNA targeting *AtLHCB1.1*, *LHCB1.2* and *LHCB1.3* CDS (GAGGACTTGCTTTACCCCGG) and *LHCB1.1*, *LHCB1.3*, *LHCB1.4*, and *LHCB1.5* CDS (GGTTCACAGATCTTCAGCGA) and the two sgRNA targeting *LHCB2.2* exon 1 (GGATTGTTGGATAGCTGATG) and *LHCB2.3* exon 1 (GATGCGGCCACCGCCATTGG) in the background of a transfer DNA insertional mutant for the *LHCB2.1* gene (SALK_005774C). The two sgRNAs targeting *LHCB1* or *LHCB2* genes were inserted into a binary vector under the control of the U6 promoter using the cloning strategy detailed (63). This binary vector contains also the Cas9 gene under the control of the synthetic EC1 promoter that is expressed only in the egg cells (64). To identify *lhcb1* and *lhcb2*, resistant plants were screened by Chl fluorescence for NPQ and photosynthetic acclimation (36), and potential candidates were further confirmed by immunoblot using antibodies against Lhcb1 and Lhcb2. For *soq1 lhcb1 lcnp*, plants were transformed by floral dipping with Agrobacterium GV3101 pSoup containing the vector pDGE277 with the four sgRNAs. Seeds from transformed plants were plated and selected on Murashige and Skoog plates with 25 μg ml^−1^ hygromycin. The hygromycin-resistant plants were selected, and the absence of LCNP was confirmed by immunoblot using an antibody raised against LCNP. Phire Plant Direct PCR kit was used for genotyping and sequencing with dilution protocol (Thermo Fisher Scientific; catalog no.: F130); primer list can be found in Table S4.

### Chl fluorescence imaging

Chl fluorescence was measured at room temperature with Walz Imaging-PAM Maxi (Fig. S3, *B* and *C*) or with SpeedZenII from JBeamBio (Figs. 4 and S4*C*). For NPQ measurements, plants or detached leaves were dark acclimated for 20 min, and NPQ was induced by 1200 μmol photons m^−2^ s^−1^ for 10 min and relaxed in the dark for 10 min. *F*_m_ after dark acclimation and throughout measurement (*F*_m_’) were recorded after applying a saturating pulse of light, which closes reaction centers, that is, blocks photochemistry. NPQ was calculated as (*F*_m_ − *F*_m_’)/*F*_m_’. *F*_v_/*F*_m_ is the maximum photochemical efficiency of PSII and is calculated as (*F*_m_ − *F*_o_)/*F*_m_, where *F*_o_ is the minimum fluorescence after dark acclimation (reaction centers are open).

### Thylakoid extraction

Thylakoid extractions were performed (65). Briefly, leaves from 6- to 8-week-old plants were ground in a blender for 30 s in 60 ml B1 cold solution (20 mM tricine–KOH [pH 7.8], 400 mM NaCl, and 2 mM MgCl_2_). Protease inhibitors are used at all steps (0.2 mM benzamidine, 1 mM aminocaproic acid, and 0.2 mM PMSF). The solution is then filtrated through four layers of Miracloth and centrifuged 5 min at 27,000*g* at 4 °C. The supernatant is discarded, and the pellet is resuspended in 15 ml B2 solution (20 mM tricine–KOH [pH 7.8], 150 mM NaCl, and 5 mM MgCl_2_). The resuspended solution is overlayed onto a 1.3 M/1.8 M sucrose cushion and ultracentrifuged for 30 min in a SW28 rotor at 131,500*g* and 4 °C. The band between the sucrose layers is removed and washed with B3 solution (20 mM tricine–KOH [pH 7.8], 15 mM NaCl, and 5 mM MgCl_2_). The solution is centrifuged for 15 min at 27,000*g* and 4 °C. The pellet is washed in storing solution (20 mM tricine–KOH [pH 7.8], 0.4 M sucrose, 15 mM NaCl, and 5 mM MgCl_2_) and centrifuged for 10 min at 27,000*g* and 4 °C. The pellet is then resuspended in storing solution. Chl concentration is measured (66). If samples are to be stored, they were flash-frozen in liquid nitrogen and stored at −80 °C at approximately 2.5 mg Chl ml^−1^. Upon using thylakoid preparation, samples are rapidly thawed and buffer is exchanged with 120 mM Tris–HCl (pH 6.8), and Chl concentration is measured. For spectroscopy experiments, thylakoids were isolated (67). For the “nontreated” condition, leaves were detached and dark acclimated overnight at 4 °C. Cold and HL treatment, followed by 5 min dark acclimation, was performed on plants prior to thylakoid extraction.

### Isolation of pigment–protein complexes

Thylakoid membranes (400 μg Chl) were solubilized at 2 mg ml^−1^ with 4% (w/v) α-dodecyl maltoside (α-DM) for 15 min on ice (solution was briefly mixed every 5 min), and unsolubilized membranes were removed by centrifugation at 14,000 rpm for 5 min. Gel filtration chromatography was performed (65) using the ÄKTAmicro chromatography system with a Superdex 200 Increase 10/300 GL column (GE Healthcare) equilibrated with 20 mM Tris–HCl (pH 8.0), 5 mM MgCl_2_, and 0.03% (w/v) α-DM at room temperature. The flow rate was 1 ml min^−1^. The proteins were detected at 280 nm absorbance.

### Protein analysis

A 5 mm diameter disc was cut from the leaf and frozen into liquid nitrogen. The leaf disc was ground with a plastic pestle, and 100 μl of sample loading buffer (62.5 mM Tris [pH 7.5], 2% SDS, 10% glycerol, 0.01% bromophenol blue, and 100 mM DTT) was added. Samples were boiled at 95 to 100 °C for 5 min and centrifuged for 3 min. From the samples, 10 μl were loaded onto a 10% SDS-PAGE gel. For the gel filtration fractions, samples were loaded at same volume from pooled adjacent fractions (three fractions for each) onto a 12% SDS-PAGE gel for immunoblot or for silver stain. After migration, the proteins were transferred to a polyvinylidene difluoride 0.45 μm from Thermo Fisher Scientific. After transferring, the membrane was blocked with Tris-buffered saline with Tween-20 (TBST) + 3% milk for 1 h followed by 1 h incubation of the primary antibody (ATP*β* AS05 085 [1:5000 dilution], Lhcb1 AS09 522 [1:5000 dilution], Lhcb2 AS01 003 [1:10,000 dilution], Lhcb3 AS01 002 [1:2000 dilution], Lhcb4 AS04 045 [1:7000 dilution] from Agrisera and rabbit antibodies against a peptide of LCNP [AEDLEKSETDLEKQ] were produced and purified by peptide affinity by Biogenes and used at a 1:200 dilution) diluted in TBST + 3% milk. The membrane was washed three times 10 min with TBST. Then incubated for 1 h with the secondary goat anti-rabbit antibody conjugated to horseradish peroxidase AS09 602 (1:10,000 dilution) from Agrisera in TBST + 3% milk. The membrane was washed three times 10 min with TBST and one time 5 min with TBS. The Agrisera ECL Bright (AS16 ECL-N-100) and Azure Biosystems c600 were used to reveal the bands.

### Clear-native PAGE analysis

Thylakoids are washed with the solubilization buffer (25 mM Bis–Tris/HCl [pH 7.0], 20% [w/v] glycerol, 10 mM sodium fluoride, and 0.2 mM PMSF) and resuspended in the same buffer at 1 mg Chl ml^−1^. An equal volume of 2% α-DM was added to the thylakoid solution for 15 min on ice in the dark. Traces of insoluble material were removed by centrifugation at 18,000*g* for 20 min at 4 °C. The Chl concentration was measured, and proteins were loaded at equal Chl content in the native gel (NativePAGE 3–12%, Bis–Tris, 1.0 mm, Mini Protein Gel, 10-well from Thermo Fisher Scientific; catalog number: BN1001BOX). Prior to loading, the samples were supplemented with sodium deoxycholate (final concentration of 0.3%). The cathode buffer is 50 mM tricine, 15 mM Bis–Tris, 0.05% sodium deoxycholate and 0.02% α-DM, pH 7.0, and anode buffer is 50 mM Bis–Tris, pH 7.0. Electrophoresis was performed at 4 °C with a gradual increase in voltage: 75 V for 30 min, 100 V for 30 min, 125 V for 30 min, 150 V for 1 h, and 175 V until the sample reached the end of the gel. The method is adapted from the study of Ref. (68).

### Pigment extraction and analysis

HPLC analysis of carotenoids and Chls was done as previously described (69). 10 μg Chl of fraction samples were extracted in 200 μl 100% acetone.

### Lipid profiling

Thylakoids or gel filtration fractions corresponding to trimers or monomers were evaporated until dryness using a vacuum evaporator, and dried samples were reconstituted in 100 μl isopropanol. Lipids were separated on Acquity Ultra Performance LC coupled to a Synapt G2 HDMS equipped with electrospray ionization source (Waters) according to an adapted protocol (70). Briefly, liquid chromatographic separation was performed on BEH C18 column (2.1 × 100 mm, 1.7 μm) using binary solvent strength gradient from 30% to 100% eluent B within 10 min at a flow rate of 0.3 ml min^−1^. Eluent A was 10 mM ammonium acetate in water:acetonitrile (60:40 v/v), and eluent B was 10 mM ammonium acetate in isopropanol:acetonitrile (90:10 v/v). The mass spectrometer was operated in positive and negative electrospray ionization, and centroid data were acquired with a mass range from 50 to 1200 Da using leucine–enkephaline for internal calibration. Lipids were identified by matching masses of molecular, typical fragments (error less than 1 mDa), and elemental compositions using isotope abundance distributions. MassLynx 4.1 was used to operate the instrument, and QuanLynx was used for peak integration (Waters Corporation). Samples were normalized by Chl content.

### Fluorescence spectroscopy on isolated thylakoids or complexes

Room temperature fluorescence emission of gel filtration fractions and dependence on step solubilization of thylakoids were performed (18) using a Horiba FluoroMax fluorimeter and Starna cells 18/9F-SOG-10 (path length of 10 mm) with Chl concentration of 0.1 μg ml^−1^. For the emission spectrum of gel filtration fractions (emission 650–800 nm with excitation at 625 nm, bandwidth, 5 nm for excitation, 5 nm for emission), samples were diluted at same absorption (Δ625–750 nm = 0.0005) in 20 mM Tris–HCl (pH 8), 5 mM MgCl_2_, and 0.03% α-DM. For the step solubilization (emission 680 nm with excitation at 440 nm, bandwidth, 5 nm for excitation, and 3 nm for emission), thylakoid preparations were diluted in 20 mM Tris–HCl (pH 8), 5 mM MgCl_2_, and two different detergents were added: first, α-DM at final 0.5% (w/v) concentration from a 10% stock solution, which dissociates the pigment-binding proteins from each other without release of Chl from their protein moiety (71), then Triton X-100 at final 5% (w/v) concentration from a 50% stock solution that denatures the pigment–proteins and yields free pigments (72). After each addition, the cuvette was turned upside down 3 to 5 times for mixing, and time for fluorescence level stabilization was allowed.

### Fluorescence lifetime measurements and fitting

Fluorescence lifetime measurements of NPQ report directly on the quenching of the Chl excited state. In contrast to yield-based measurements, fluorescence decay–based measurements are not affected by nonquenching processes that can decrease the fluorescence yield such as pigment bleaching or changes in concentration, scattering of light, or chloroplast movement/shielding. Method used is adapted for fluorescence lifetime snapshot (34). Time-correlated single photon counting was performed on detached leaves, isolated thylakoids, and gel filtration fractions. Excitation at 420 nm was provided by frequency doubling the 840 nm output of a Ti:sapphire oscillator (Coherent, Mira 900f, 76 MHz). The laser intensity was ∼18,000 μmol photons m^−2^ s^−1^/pulse (∼20 pJ/pulse), sufficient to close reaction centers. Emission was monitored at 680 nm using an MCP PMT detector (Hamamatsu; R3809U). The full width at half maximum of instrument response function was ∼30 to 40 ps.

It has been shown that a wide range of exponentials can reasonably fit any ensemble fluorescence decay measurement (49), with no easy way to distinguish between the different “models.” Therefore, to gain a simple unbiased description of the fluorescence dynamics in each sample, each decay was fit to a triexponential model (PicoQuant; FluoFit Pro-4.6) without constraining any specific kinetic component, and an amplitude-weighted average fluorescence lifetime (τ_avg_) was calculated. Representative decays and fits are shown in Fig. S10. The extent of quenching was then evaluated by comparison of τ_avg_ values from nontreated and cold HL-treated plants, quantified as $NPQ\tau =\frac{\tau_{\mathrm{avg},\mathrm{nontreated}}-\tau_{\mathrm{avg},\mathrm{cold}\;\mathrm{HL}-\mathrm{treated}}}{\tau_{\mathrm{avg},\mathrm{cold}\;\mathrm{HL}-\mathrm{treated}}}$. Prior to each measurement, qE was relaxed by dark acclimation for at least 5 min.

## Data availability

The authors declare that all data supporting the findings of this study are included in the article and its supporting information file and are available from the corresponding author upon request. Source data for all figures are provided with the article. Sequence data from this article can be found in the Arabidopsis Genome Initiative under accession numbers At1g29920 (Lhcb1.1), At1g29910 (Lhcb1.2), At1g29930 (Lhcb1.3), At1g56500 (SOQ1), At2g05070 (Lhcb2.2), At2g05100 (Lhcb2.1), At2g34420 (Lhcb1.5), At2g34430 (Lhcb1.4), At3g27690 (Lhcb2.3), At3g47860 (LCNP), At4g31530 (ROQH1), and At5g54270 (Lhcb3).

## Supporting information

This article contains supporting information.

## Conflict of interest

The authors declare that they have no conflicts of interest with the contents of this article.
